# *FGFR2* Is Amplified in the NCI-H716 Colorectal Cancer Cell Line and Is Required for Growth and Survival

**DOI:** 10.1371/journal.pone.0098515

**Published:** 2014-06-26

**Authors:** Anjili Mathur, Christopher Ware, Lenora Davis, Adi Gazdar, Bo-Sheng Pan, Bart Lutterbach

**Affiliations:** 1 Merck Research Labs, Boston, Massachusetts, United States of America; 2 Hamon Center for Therapeutic Oncology, University of Texas Southwestern, Dallas, Texas, United States of America; Florida International University, United States of America

## Abstract

Aberrant kinase activation resulting from mutation, amplification, or translocation can drive growth and survival in a subset of human cancer. *FGFR2* is amplified in breast and gastric cancer, and we report here the first characterization of *FGFR2* gene amplification in colorectal cancer in the NCI-H716 colorectal cancer cell line. *FGFR2* is highly expressed and activated in NCI-H716 cells, and FGFR selective small molecule inhibitors or FGFR2 shRNA strongly inhibited cell viability *in vitro*, indicating “addiction” of NCI-H716 cells to FGFR2. NCI-H716 growth in a xenograft model was also inhibited by an FGFR small molecule inhibitor. FGFR2 was required for activation of multiple downstream signaling proteins including AKT, ERK, S6RP and NFKB. Inhibition of downstream kinases such as AKT or ERK alone had modest effects on proliferation, whereas combined inhibition of AKT and ERK signaling resulted in a loss of viability similar to FGFR2 inhibition. We identified elevated *FGFR2* expression in a small subset of primary colorectal cancer, however *FGFR2* amplification was not observed. Although *FGFR2* amplification is not common in primary colon cancer or lymph node and liver metastases, other subsets of colorectal cancer such as ascites, from which the NCI-H716 cell line was derived, have yet to be tested. These results suggest that emerging FGFR inhibitor therapeutics may have efficacy in a subset of colon cancer driven by *FGFR2* amplification.

## Introduction

Advanced, late stage colorectal cancer is associated with significant mortality and remains an unmet medical need. Early diagnosis results in a highly favorable prognosis, such that stage 1 and stage 2 disease have an 80–90% five year survival. By contrast stage 3 and stage 4 metastatic disease are associated with five year survival of 60% and 8%, respectively [1]. Genetic aberrations arising in early stage disease include *APC* mutations, while *KRAS, BRAF, p53*, and *PIK3CA* mutations are found in later stages of tumor development [2]–[4]. However, these gene mutations have not impacted treatment of colorectal cancer because they are either loss of function mutations (*APC, p53*) or are not responsive to MEK or PI3K inhibitors (*BRAF, KRAS, PIK3CA*). Although tyrosine kinase amplification, translocation, or mutations are not common in colorectal cancer, *EGFR* amplification can be found in a subset of colorectal cancers, [5], [6]. EGFR inhibitory antibodies such as Cetuximab can lead to responses and survival benefit in *KRAS* wildtype cancers, but the role of *EGFR* amplification is not clear [7]. *Erbb2* amplification has also been reported [6], [8], and can be associated with response to Trastuzumab [9].

*FGFR2* amplification has been described in 5% of gastric cancers [10] and 1–4% of breast cancers [11], [12], but has not been reported in colon cancer. Breast and gastric cancer cell lines harboring *FGFR2* amplification are highly sensitive to *FGFR2* inhibitors in preclinical models [13]–[15], and amplification in both breast and gastric cancer is strongly associated with poorly differentiated, late stage tumors [11], [16]. In gastric cancer *FGFR2* amplification can occur in a metastatic tumor but not in the associated primary tumor, also consistent with a role in metastatic and late stage cancer [16], [17].

Here we define a novel *FGFR2* amplification in the NCI-H716 colorectal cancer cell line that was derived from the ascites of a poorly differentiated colon adenocarcinoma. *FGFR2* gene amplification results in FGFR2 overexpression and constitutive activation. Importantly, NCI-H716 cell growth and survival *in vitro* and in a murine xenograft model were dependent on FGFR2. Immunohistochemistry revealed *FGFR2* overexpression in a subset of primary colon cancer, but we did not observe amplification. However, these arrays did not contain ascites-derived samples representative of the origin of NCI-H716 cells. Although *FGFR2* amplification and overexpression are rare in colorectal cancer, our *in vitro* and *in vivo* models suggest that emerging FGFR inhibitors could have efficacy in a subset of colorectal cancer harboring this amplification.

## Materials and Methods

### Cell lines and reagents

Cell lines were from American Type Culture Collection (ATCC) and were maintained in RPMI plus 10% fetal calf serum and 100 ug/ml Pennicillin/Streptavidin (Sigma). PD173074 was from Sigma (St Louis, MO). MK2461 was from Merck [18].

### FISH analysis

DNA FISH was performed on NCI-H716 cells treated with colcemid (0.02 µg/ml for 3 hours) and on microarray tissue cores as described previously [19], [20] using bacterial artificial chromosome clones RP11-62L18 for *FGFR2* probes. This FISH probe contains the genomic sequence of Chr10: 123,224,100–123,398,498, which encompasses the entirety of the FGFR2 gene at Chr10: 123,237,844–123,353,481. Probes were labeled directly using Spectrum Orange dUTP and Spectrum Green dUTP (Abbott Molecular Inc., Des Plaines, IL).

### Immunohistochemistry

H80 antibody (Santa Cruz Biotechnology, Santa Cruz, CA) was used at a dilution of 1∶50 on NCI-H716 xenograft and Asterand gastric cancer sections and at a 1∶20 dilution on array cores from US Biomax Inc (Rockville, MD): CO802 (78 primary tumors, 2 normal), CO702 (69 samples–30 primary tumors, 25 lymph nodes, 8 liver metastases, 9 normal), CO992 (33 primary tumors and 33 lymph node metastases), BCO5115 (69 total, 60 primary tumors, 7 liver metastases), In these arrays, 20 patients were below 35 years of age. Staining was performed on a Ventana Discovery XT using Rabbit Ultra-HRP. Detection was with ChromoMap kit (Ventana Molecular Discovery Systems, Tucson, AZ).

### ShRNA production and infection

shRNA sequences were:

F1: GCCAACCTCTCGAACAGTATTCAAGAGATACTGTTCGAGAGGTTGGC,

F2: GGACTTGGTGTCATGCACCTTCAAGAGAGGTGCATGACACCAAGTCC

F3: GGACTGTAGACAGTGAAACTTCAAGAGAGTTTCACTGTCTACAGTCC

F4: GAGATTGAGGTTCTCTATATTCAAGAGATATAGAGAACCTCAATCTC

Luciferase: CACCGGTGTTGTAACAATATCGACGAATCGATATTGTTACAACACC

AAA. Scrambled: CACCGTCTCCACGCGCAGTACATTTCGAAAAATGTACTGCGCGTG

GAGACAAAA Oligos were annealed and 5′BbsI and 3′ SpeI used to clone into a proprietary ENTR plasmid followed by conversion to Plenti6/Block-iT-DEST (Invitrogen, Grand Island NY) using Gateway (Invitrogen, Grand Island NY). Virus production and titer determination were as directed by Invitrogen. For growth analysis, cells were seeded at 4000 cells/well in 96 well plates, while for western analysis cells were seeded at 40,000 cells in 12 well plates. Viral supernatants were added for 20 hours in the presence of 8 ug/ml polybrene, at which point viral supernatants were removed and replaced with growth medium containing 10% fetal bovine serum. Lysates were prepared for western analysis 48 hours later.

### Compound treatment and growth assays

PD173074 (Sigma, St Louis MO) and MK2461 were diluted in DMSO to create a titration series as previously described [14]. Gleevec, Lapatinib, PHS665753, and PD168393 were from ChemieTek (Indianapolis IN) Anti IGF1R antibody AF305 was from R/D systems. Cells were treated in triplicate wells, and after 3 days cell numbers were quantitated using the Vialight reagent (Vialight assay kit, Cambrex, Rockland ME). Luminescence was quantified with a Topcount NXT HTS (Perkin Elmer, Waltham, MA) and IC50 determinations made by using logistic 4 parameter curve fitting. For quantitation of phosphotyrosine in FGFR2 and blots were scanned and quantitated using ImageQuant software. Values corresponding to band intensity were plotted against drug concentration to establish an IC50 of drug inhibition. ScanMAX profiling of 442 kinases was at Ambit Biosciences (San Diego, CA).

### Western blotting, immunoprecipitation, antibodies, and growth factors

Lysates were prepared in 30 mmol/L Tris-HCL, pH 7.5, 50 mmol/L NaCl, 5 mmol/L EDTA, 50 mmol/L NaF, 30 mmol/L NaPPi, 1% Triton, 0.5% IGEPAL, 10% Glycerol, 1 mmol/L Vanadate, 1 mmol/L bpPhen (Calbiochem), and protease inhibitor (Roche) and western blotting and immunoprecipitation were as described [21], [22]. Antibodies against FGFR2 included N-terminus MAB6841 (R&D Systems, Minneapolis, MN) and H80 (Santa Cruz), C-terminus C-20 (Santa Cruz) and Y653/654 specific 3471 ((Cell Signaling Technology, (CST), Danvers MA.)). Additional antibodies from CST were: pAKT S473 (4060), AKT (9272), pERK (4370), ERK (9102), pS6RP S235/236 (2217), S6RP (2211), cleaved PARP (9542), beta actin (4967), p105 NFKB S933, p105 NFKB, GAB1 Y672, FRS2 Y436, SHC Y317 CRKII Y221, PRAS40 T246, PDK1 S241, GSKIII S9, PKC S660, RSK S227 (PDK1 site), RSK S359/363 (ERK site), AMPK T172 (LKB site) and GAPDH. 4G10 phosphotyrosine antibody was from Upstate (Charlottesville VA). Recombinant FGF2 was from R&D Systems (Minneapolis MN).

### Flow cytometry

A Becton Dickinson FACS Calibur was used for flow cytometry on NCI-H716 cells. Cells were fixed overnight in 1 ml ice cold 70% ethanol then washed in PBS and stained with PI/RNAse (BD Pharmingen, San Diego) for 3 hours. ModFit software was used to determine relative distribution in G1, S, G2/M, and manual gating to determine subG1 content.

### Xenograft

Animal studies were conducted according to IACUC (Institutional Animal Care and Use Committee) guidelines and experiments were approved by Merck research labs ethics review committee. Systematic monitoring and recording of welfare of mice was according to ARRIVE guidelines for appearance, size, coat condition, posture, gait, activity levels, interaction with the environment and clinical signs. Euthanasia was coducted by expose of animals to CO_2_ until complete cessation of breathing was observed for a minimum of 2 minutes (a total of approximately 5 to 10 minutes). Animals were visually inspected for the absence of movement and respiration. Death was also ensured by removal of tumor tissue or multiple organs.

5×10^6^ NCI-H716 cells were implanted subcutaneously into BALB/c nude mice. After reaching 200 mm^3^, tumors were treated with vehicle, 300 mg/kg or 800 mg/kg, once daily for 24 days. 10 mice were in each group, and tumor size was determined by caliper measurement. No animals experienced greater than 5% weight loss during treatments. FGFR2, AKT, and ERK inhibition *in vivo* was measured by dosing animals and collecting tumors at 4 and 24 hours post treatment. Tumors were placed in liquid nitrogen and processed by disruption in 600 ul lysis buffer in a tissue lyzer (Qiagen). Lysates were quantitated and processed for SDS-PAGE and western blotting as described for cell lysates.

## Results

### *FGFR2* is amplified, and activated in NCI-H716 cells

**Figure 1:**
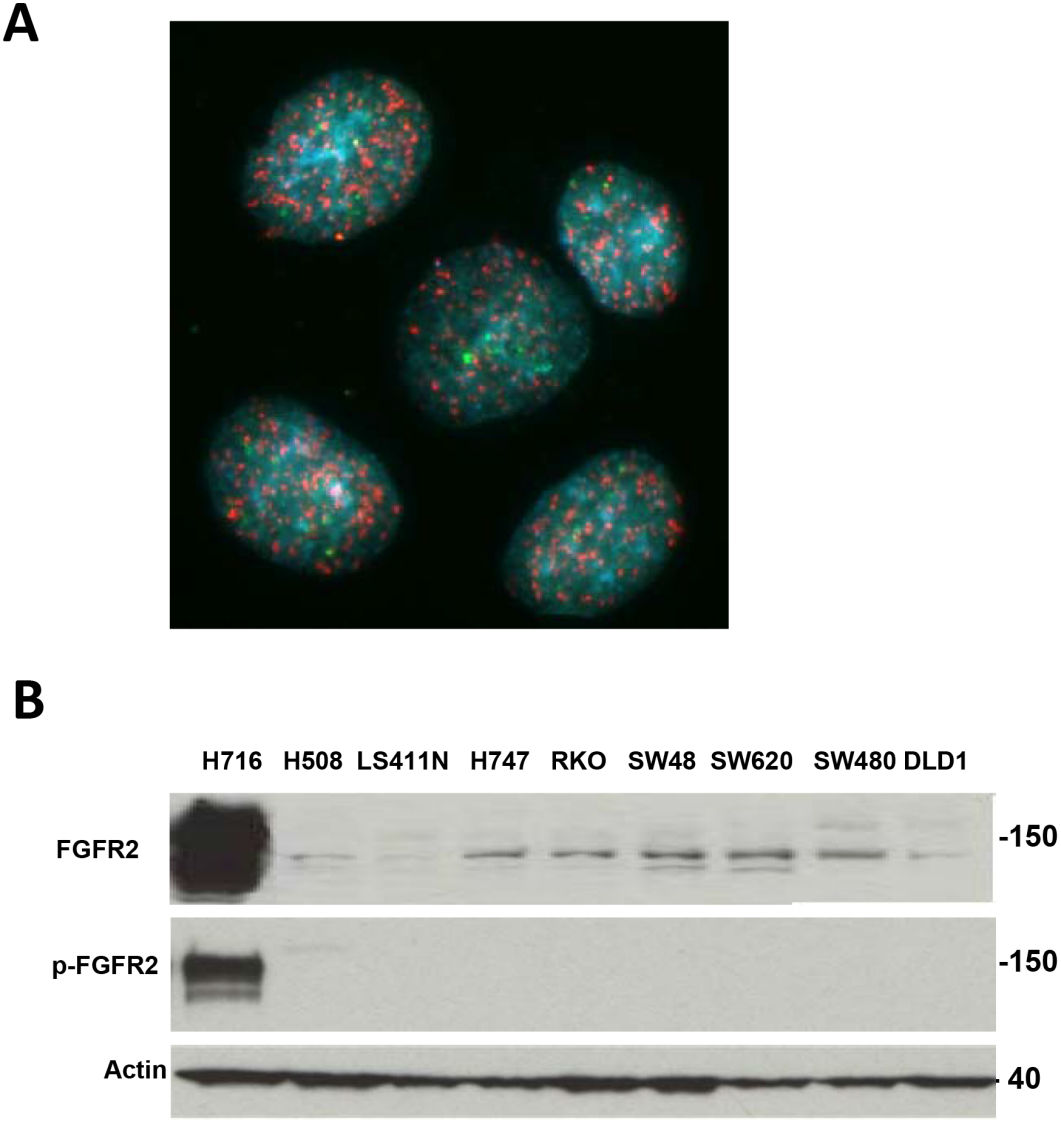
*FGFR2* is amplified, overexpressed, and activated in NCI-H716 cells. A. NCI-H716 cells were treated with colcemid (0.02 µg/ml for 3 hours), fixed with methanol/acetic acid, and dropped onto a microscope slide according to materials and methods. Bacterial artificial chromosome clone RP11-62L18 was labeled with Spectrum Orange dUTP and a centromere probe was labeled with Spectrum Green dUTP (Abbott Molecular Inc., Des Plaines, IL) and hybridized to fixed cells. Red indicates *FGFR2* and green indicates Centromere. B. FGFR2 is overexpressed and contains high levels of tyrosine phosphorylation in the NCI-H716 cell line. A, Cell lysates (prepared according to Materials and Methods) from untreated or FGF2 treated (30 ng/ml, 5 minutes) cells were lysed and protein concentration was determined with BCA kit (Pierce Thermo Fisher Rockford Ill). Equal lysate amounts (50 ug) were subjected to SDS-PAGE and western blotting with FGFR2 antibodies made against the N terminus (H80. MAB6841), C terminus (C20) and activation loop phosphorylation Y653/654 (3471, p-FGFR2) and Actin.

Because *FGFR2* amplification can drive gastric and breast cancer cell growth, we searched for additional cell lines that harbor similar *FGFR2* copy gain. DNA microarrays confirmed known *FGFR2* amplification in KATOIII and SNU16 gastric cancer cell lines [23], [24] and identified a novel highly focal amplification in the NCI-H716 colorectal cancer cell line (Figure S1A in File S1. The Oncomine database [25] also revealed high *FGFR2* messager RNA expression in NCI-H716 cells. Fluorescent in situ hybridization (FISH) revealed striking *FGFR2* copy gain and gene duplication in homogeneously staining regions (Figure 1A). The green centromere probe did not reveal copy gain, consistent with the highly focal amplicon at the *FGFR2* locus (Figure S1A in File S1). A FISH probe for *Met* revealed no copy gain in NCI-H716 at this chromosome 7 locus, and the *FGFR2* probe did not show amplification in DLD1 colon cancer cells lacking *FGFR2* copy gain (data not shown). We then compared *FGFR2* expression and activation in NCI-H716 cells relative to a panel of nine colorectal cancer cell lines without *FGFR2* amplification, and we found striking FGFR2 overexpression and phosphorylation only in NCI-H716 cells (Figure 1B). FGF2 did not further activate FGFR2 in NCI-H716 cells, revealing that the receptor is maximally phosphorylated (data not shown). The level of FGFR2 activation in NCI-H716 cells was similar to levels seen in the *FGFR2* amplified SNU16 gastric cancer cell line (Figure S2 in File S1). We conclude that in our panel of colon cancer cell lines that *FGFR2* is highly overexpressed and activated only in NCI-H716 cells.

### FGFR2 is required for growth of NCI-H716 cells

**Figure 2:**
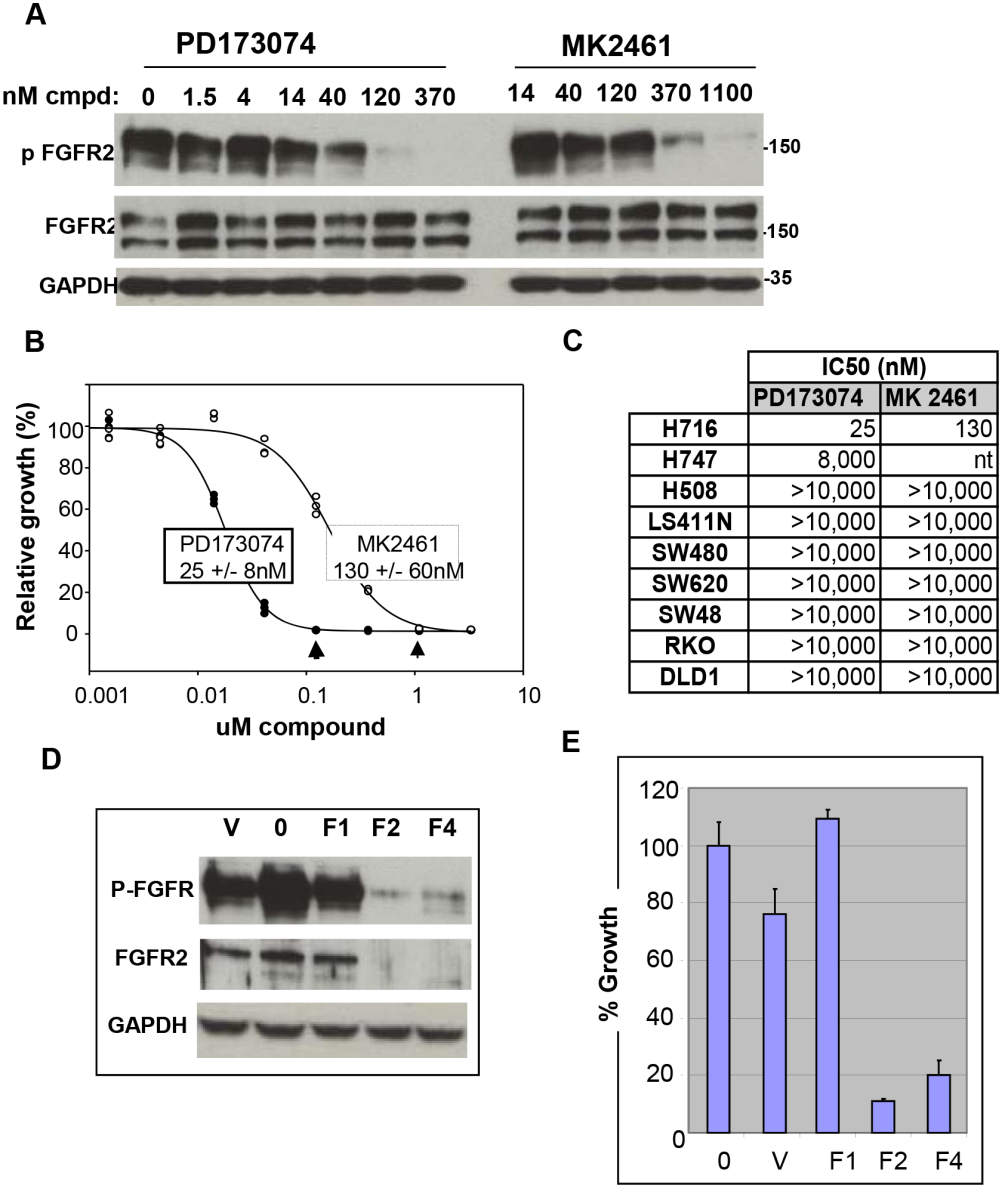
FGFR2 is required for growth of NCI-H716 cells. A. PD173074 and MK2461 inhibit FGFR2 phosphorylation. Cells were treated for 1 hour with a titration of PD173074 as described in Materials and methods. Lysates were prepared and 50 ug protein was subjected to SDS-PAGE and western blotting with phospho Y653/654 FGFR and MAB6841 total FGFR2 antibody. B. PD173074 and MK2461 inhibit NCI-H716 cell growth. Cell lines were plated at 4000 cells/well and incubated overnight. NCI-H716 cells were treated with a titration of PD173074 as described in Materials and Methods. Cell growth was measured with Vialight reagent, and growth was presented relative to untreated cells. C. PD173074 and MK2461 selectively inhibit growth of NCI-H716 cells. Colon cancer cell lines listed were plated at 4000 cells/well and 24 hours later were treated with a titration of compounds. 4 days later cell growth was measured with vialight and IC50s were calculated from graph pad prism. D. *FGFR2* shRNA decreases FGFR2 protein in NCI-H716 cells. *FGFR2* shRNA was prepared and NCI-H716 cells were infected as described in methods. Left, FGFR2 expression was analyzed with phospho Y653/654 and total protein with MAB6841. E. Growth was analyzed 5 days post infection with Vialight reagent.

*FGFR2* amplification and activation in NCI-H716 cells suggested this kinase may be required for cell growth. We treated NCI-H716 cells with two FGFR small molecule inhibitors to test a role for FGFR2 in NCI-H716 cell growth. In a large panel of kinses, we found PD173074 to be a highly selective inhibitor of FGFR-1,-2,-3 [14], and we further confirmed the remarkable selectivity here with an separate large panel of kinases (Figure S3 in File S1). We also treated cells with MK2461, a Met kinase small molecule inhibitor that also inhibits FGFR2 phosphorylation and growth of *FGFR2* amplified gastric and breast cancer cell lines [13]. PD173074 and MK2461 potently inhibited FGFR2 phosphorylation in NCI-H716 cells (Figure 2A) with an IC50 of 18 nM (PD173074) and 165 nM (MK2461, Figure S4A in File S1). Both compounds potently inhibited NCI-H716 growth with IC50 values of 25 nM for PD173074 and 130 nM for MK2461 (Figure 2B). Importantly, the IC50 for growth inhibition and FGFR2 phosphorylation inhibition were similar, and both values were also similar to values previously obtained for SNU16 and KATOIII cell lines [13], [14]. Having observed potent inhibition of NCI-H716 cell growth, we next tested both compounds for inhibition in a panel of colon cancer cell lines. Both FGFR inhibitor compounds displayed a striking selectivity for inhibition of NCI-H716 cells, with greater than 80-fold (MK2461) or 400-fold (PD173074) lower IC50 relative to non-*FGFR2* amplified colon cell lines (Figure 2C). The FGFR inhibitor AZD4547 also selectively inhibited growth in NCI-H716 cells (data not shown). Finally, NCI-H716 growth was not inhibited by treatment with kinase inhibitor compounds lacking FGFR inhibition, including Tarceva, Lapatinib, Gleevec, PHA665752 (a Met inhibitor) PD168393 (an irreversible EGFR inhibitor) and an IGF1R inhibitory antibody (Figure S4B in File S1).

We further confirmed a critical role for FGFR2 in NCI-H716 cell growth using *FGFR2* shRNA. We first identified two hairpins (F2 and F4, Figure 2D) with efficient *FGFR2* knockdown. These shRNA hairpins caused potent growth inhibition in NCI-H716 cells (Figure 2E). By contrast, control shRNA and shRNA that did not decrease FGFR2 protein levels (V and F1) did not inhibit growth of NCI-H716 cells (Figure 2D, E).

### Multiple signaling proteins including AKT, ERK and NFKB are activated by FGFR2

**Figure 3:**
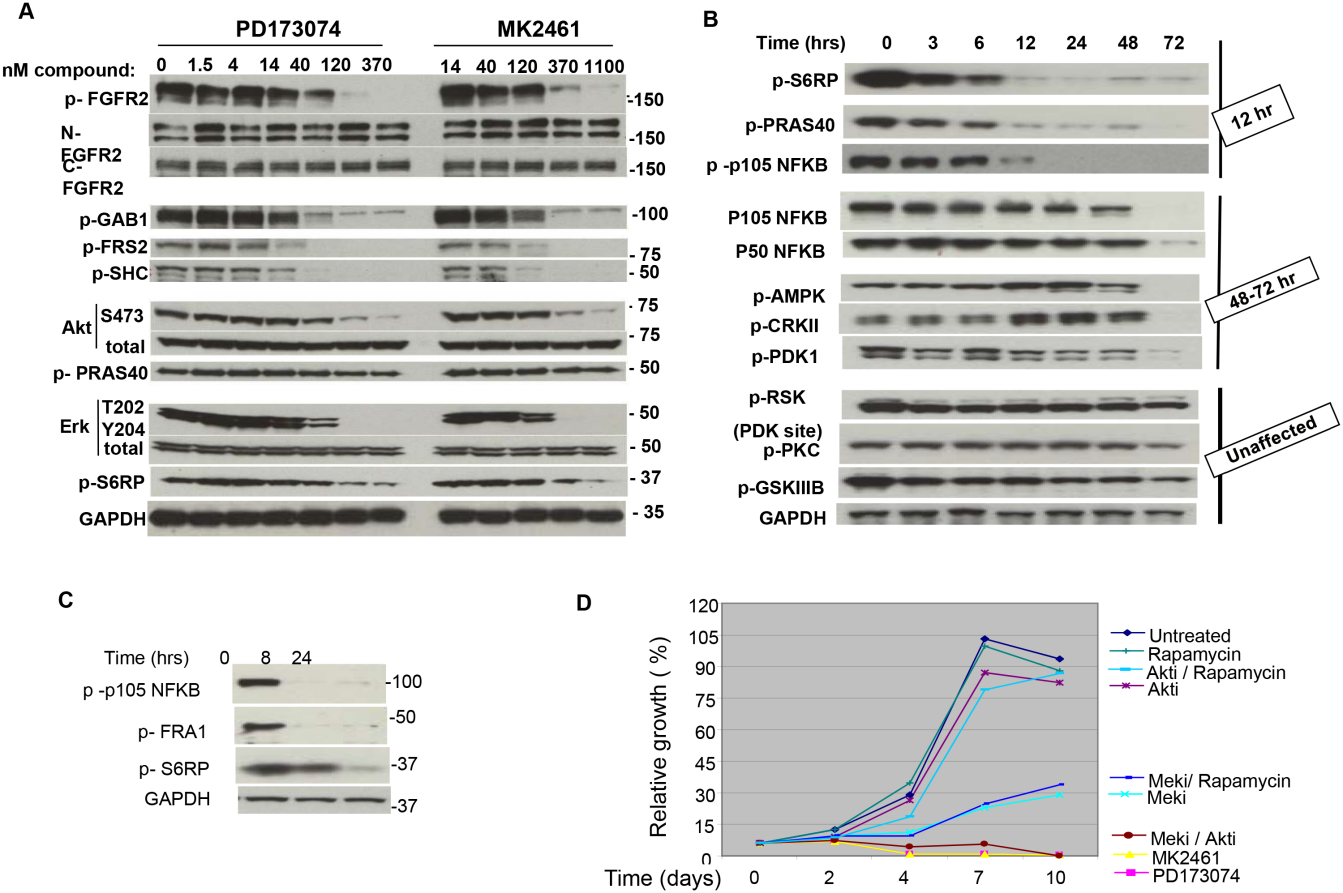
FGFR2 activates multiple signaling pathways in NCI-H716 cells. A. Lysates used in Fig. 2A were analyzed by western blotting for phosphorylated or total proteins after a 2 hour treatment with indicated compound titration. “p” indicates phosphoprotein, and the phosphorylation sites are listed in Methods. B. Time course of inhibition for multiple signaling proteins. NCI-H716 cells were treated with 100 nM PD173074 for the indicated times and signaling pathways were analyzed by SDS PAGE and western blotting. 100 nM PD173074 was selected because this amount caused full inhibition of pERK at the 2 hour time point. “p” indicates phosphoprotein, and the phosphorylation sites are listed in Methods. Terminology on the right side of figure groups proteins according to the time at which inhibition or protein loss occurs. C. NCI-H716 cells were treated with 100 nM PD0325901 for the indicated time. Lysates were prepared for SDS PAGE and western blotting with the indicated antibodies. D. NCI-H716 cells plated at 4,000 cells/well were treated 24 hours later with 1 uM L-547 (AKTi), 100 nM PD0325901 (MEKi), 5 nM Rapamycin (rapamycin), 100 nM PD173074, or 1.5 uM MK2461, or the indicated combinations. After 5 days compound and media were removed and replaced with fresh compound and media. After an additional 5 days (10 day total assay) relative cell growth was determined using Vialight reagent.

**Figure 4:**
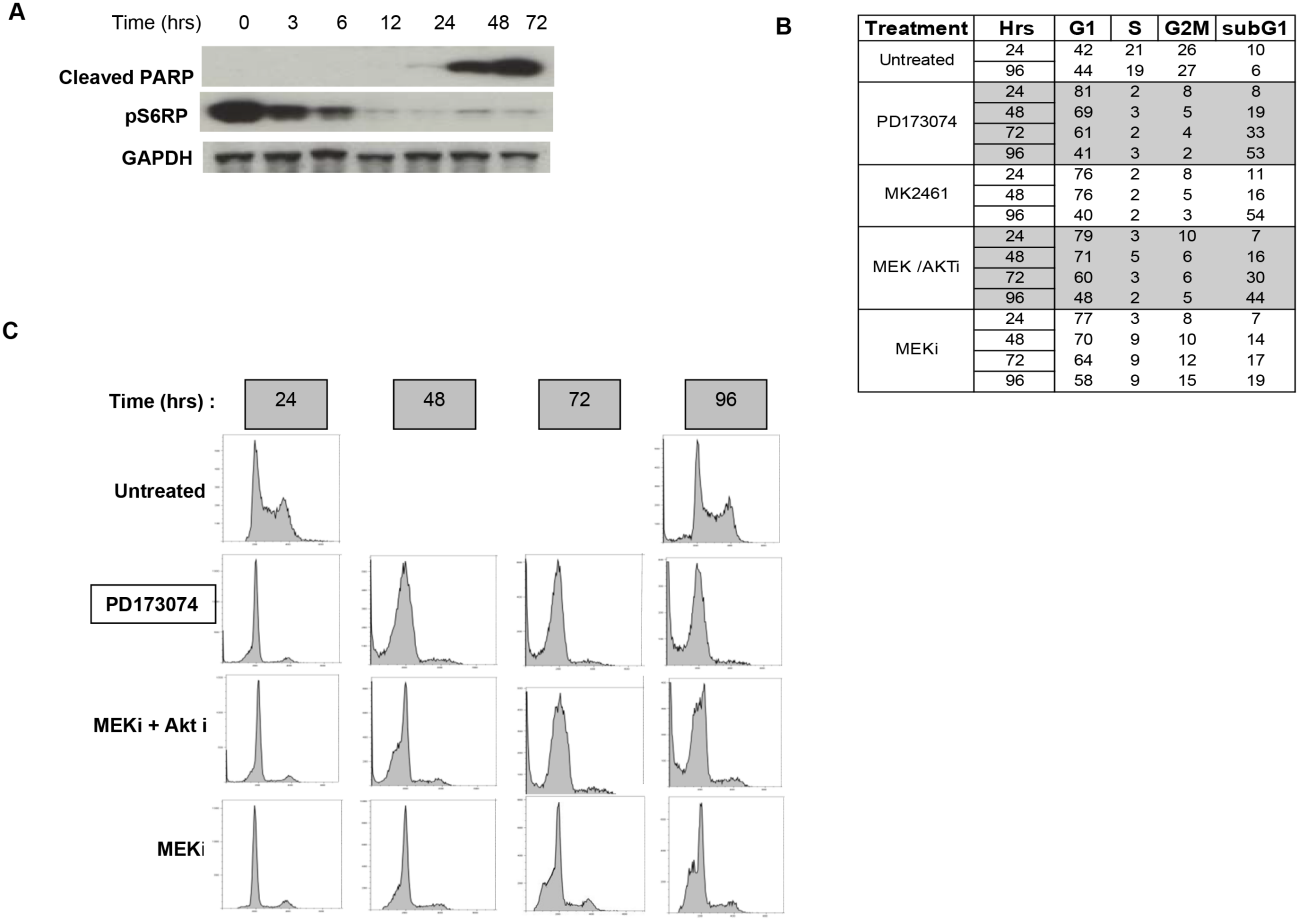
FGFR2 inhibition causes cell death in NCI-H716 cells. A. Lysates from Figure 3B reveal increased PARP cleavage. Phospho S6RP is included as reference and GAPDH was used as a loading control. B. Cell cycle profile of treated cells. 1×10exp6 NCI-H716 cells were treated with 100 nM PD173074, MEK inhibitor (100 nM) or MEK + AKT inhibitor (L-547, 1 uM) or DMSO (untreated) were isolated at the indicated time points and processed for Propidium Iodide staining and FACS analysis as described in Materials and methods. B. indicates a tabular representation of cell cycle profiles as determined by manual gating for G1, S, G2/M and subG1 areas. C is the cell cycle profiles.

We previously reported that *FGFR2* activated AKT and ERK signaling pathways in FGFR2 amplified gastric cancer cell lines [14]. We therefore defined the FGFR2 signaling network in NCI-H716 cells by analyzing phosphorylation of multiple adaptor proteins and signaling pathways after FGFR2 inhibition. After two hours of treatment with a titration of MK2461 and PD173074 we observed loss of phosphorylation in kinase adaptor proteins GAB1/2, FRS1/2, and SHC. We also observed loss of ERK and AKT phosphorylation (Figure 3A). Interestingly, targets downstream of AKT and ERK such as PRAS40 and S235/236 in S6RP (and S9 in GSKIII, data not shown) remained phosphorylated at this 2 hour timepoint. We next anlayzed inhibition of AKT and ERK targets at later time points after treatment with 100 nM PD173074. We also examined several other pathways to determine if delayed kinetics of inhibition was also occurring. In contrast to the 2 hour timepoint, both S6RP and PRAS40 phosphorylation were strongly inhibited 12 hours after compound addition (Figure 3B). We also found a similar delayed inhibition of serine-933 phosphorylation in NFKB p105, suggesting that NFKB signaling is part of NCI-H716 growth (Figure 3B). NFKB P50 and P105 protein levels were decreased only at 72 hours. While inhibition 12 hours post treatment may result from an extended half life of the signaling proteins, inhibition at later time points such as 72 hours may be secondary to growth inhibition (and cell death as described below in Figure 4). CRKII Y221, AMPK T172, and PDK1 S241 were inhibited only at 48–72 hours post treatment, again suggesting that loss of these sites may be secondary to growth inhibition. CRKII, AMPK, and PDK protein levels did not decrease during the time course (data not shown). Finally, other sites such as GSKIII S9 (an AKT phosphorylation site), PKC S660, RSK S227 (a PDK1 phosphorylation site) remained phosphorylated even after 72 hours (Figure 3B). This result reveals that PDK1 remains active and able to phosphorylate target sites despite cell growth inhibition. Although the S227 PDK1 phosphorylation site on RSK was not inhibited, the ERK phosphorylation site serine-359/363was inhibited within 2 hours of PD173074 treatment (Figure S5 in File S1). GSKSIII Serine-9 also remained phosphorylated at later time points, and it may be possible that other kinases apart from AKT maintain phosphorylation at this site. Finally, because MEK inhibition impacted NCI-H716 growth, we further defined the MEK signaling network using 100 nM of the allosteric MEK inhibitor PD0325901 [26]. Serine-933 in p105 NFKB and serine-265 in FRA1 are described as ERK phosphorylation sites and we confirmed loss of these sites. Serine-235/236 in S6RP is also a MEK target in NCI-H716 cells (Figure 3C). We conclude that FGFR2 inhibition resulted in a biphasic pattern of pathway inhibition, with ERK and AKT inhibited at early time points, while PRAS40, S6RP, and transcription factors such as NFKB p105 showed a delayed inhibition. Other signaling proteins such as PDK1 and PKC isoforms remained phosphorylated even at later time points. However it remains possible that despite phosphorylation proteins such as PKC isoforms may be inactive due to other factors such as cellular mislocalization. Overall these results caution that analysis of a signaling network only at early timepoints after inhibitor treatment may not fully define the range of signaling effectors.

### Combined inhibition of AKT and ERK is required to fully inhibit NCI-H716 growth

In NCI-H716 cells ERK, AKT and S6RP phosphorylation requires FGFR2, and we next used selective allosteric inhibitors of MEK, AKT, and mTor to define the role of these pathways in NCI-H716 growth. Although MEK inhibitor PD0325901 decreased NCI-H716 growth with an IC50 of 60+/−14 nM, a time course indicated that NCI-H716 cells remained in progressive growth (Figure 3D, turquoise line). We found that 1 uM AKT inhibitor L-547 or 5 nM rapamycin alone or in combination had only minor effects on growth despite strong inhibition of AKT and S6RP phosphorylation (Figure S6 in File S1). By contrast, a combination of AKT inhibitor and MEK inhibitor blocked growth and caused a decrease in cell number similar to FGFR inhibition (Figure 3D). Therefore both AKT and ERK are critical effectors of FGFR2 in NCI-H716 cells. By contrast, a combination of MEK inhibition and rapamycin was not superior to MEK inhibition alone. This is consistent with our biochemical results (Figure 3C) that S6RP is downstream of MEK in this cell line, so that ERK inhibition already leads to inhibition of S6RP. In addition, the lack of a phenotype with rapamycin suggests that multiple effectors of ERK must be inhibited in combination to mimic the growth inhibition observed with MEK inhibitor.

### Apoptosis is the mechanism for growth inhibition

The starting cell number was reduced in cells treated with PD173074 and with the AKT and MEK inhibitor combination, suggesting these inhibitors caused cell death. Cleaved PARP, a marker of apoptosis, was strongly induced after PD173074 treatment (Figure 4A) and with MK2461 (not shown). Flow cytometry and cell cycle content revealed growth arrest after 24 hours of treatment with FGFR inhibitors or MEK/AKT inhibitor combinations as seen in the loss of S phase population (Figure 4B, C) At 48 hours and later time points the growth arrest was followed by prominent induction of a subG1 population. In fact at 4 days post treatment the subG1 fraction represented half of the cell population. By contrast, MEK inhibition decreased cells in S phase at 24 hours, but neither eliminated S phase cells at later time points nor caused similar prominent cell death (Figure 4B, C). Finally, images of NCI-H716 cells reveals widespread cell fragmentation after 72 hours of treatment with PD173074 or 1 uM MK2461, consistent with cell death (Figure S7 in File S1). These results reveal that NCI-H716 cell survival is dependent on FGFR2, and also support prior evidence that both AKT and ERK activity are necessary for survival.

### FGFR2 is required for NCI-H716 xenograft tumor growth

**Figure 5:**
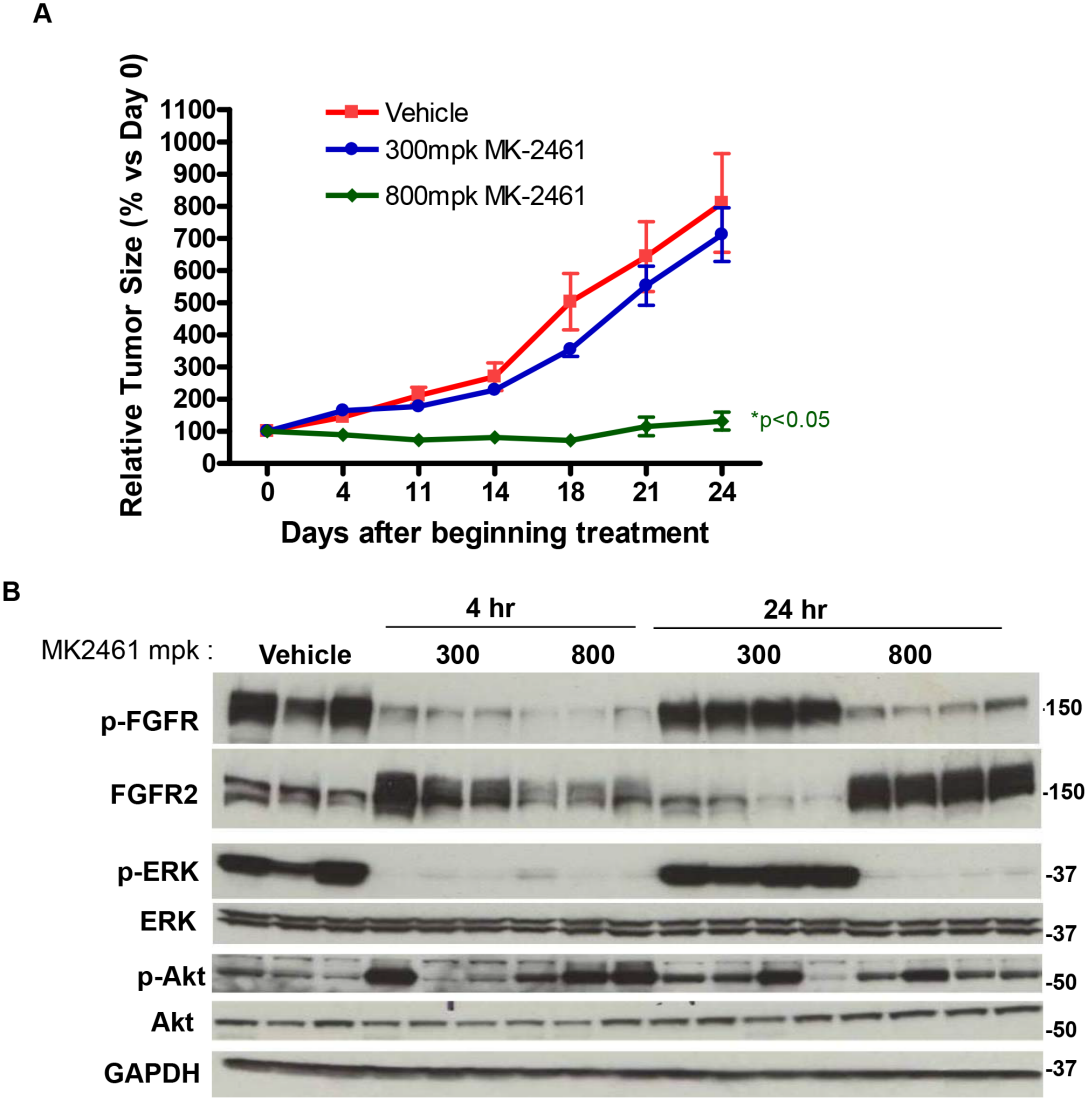
MK2461 has efficacy in NCI-H716 xenografts. A. 5×10exp6 NCI-H716 cells were implanted subcutaneously into Balb/c nude mice and treated with MK2461 at either 300 mg/kg or 800 mg/kg on a QD dosing schedule. Relative tumor growth is indicated on the Y axis. B. Tumor bearing mice were treated with a single dose of MK2461 at either 300 mg/kg or 800 mg/kg. Tumors were isolated at either 4 or 24 hours post treatment, and placed in liquid nitrogen. Tumors were processed using the tissue lyzer (Qiagen) as described in materials and methods and analyzed by SDS-PAGE and western blotting with the indicated phospho and total antibodies.

Potent NCI-H716 sensitivity to FGFR inhibitors *in vitro* suggested that xenograft tumor growth might also be inhibited *in vivo*. NCI-H716 was derived from an ascites, and consistent with adaptation to this environment, the cell line proliferates unattached in suspension. We therefore attempted to create an ascites model using luciferase expressing NCI-H716 cells injected into the peritoneal cavity. However luciferase imaging revealed a lack of tumor cell expansion (data not shown). We therefore tested for tumor growth inhibition in a standard subcutaneous xenograft model. Because PD173074 has poor pharmacokinetic properties, we treated mice with MK2461. When tumors reached 200 mm^3^ in size, MK2461 was dosed once daily (QD) at either 300 or 800 mg/kg. During the 21 day study, the 800 mg/kg treatment caused full inhibition of tumor growth, while 300 mg/kg resulted in partial inhibition that was not statistically significant (Figure 5A). Neither dose resulted in body weight loss exceeding 5% (data not shown). Both high and low doses of MK2461 caused full inhibition of FGFR2, ERK, and AKT phosphorylation in tumors 2 hours after dosing (Figure 5B). However, at 23 hours after dosing, only the 800 mg/kg dose had significant inhibition of FGFR2 and ERK phosphorylation. The inability of the 300 mg/kg dose to cause continuous inhibition of FGFR2 and ERK signaling may explain the lack of associated efficacy. Although 800 mg/kg caused full growth inhibition, no cleaved PARP was observed *in vivo*, in contrast to *in vitro* treatment with MK2461 (data not shown). The lack of cleaved PARP *in vivo* is also consistent with the absence of regression in the xenograft model, and may result from a lack of potent inhibition of AKT phosphorylation. For example, when only ERK was robustly inhibited *in vitro* (with MEK inhibitor PD0325901), there was decreased growth but not cell death. Therefore the lack of potent AKT inhibition may explain why regression was not observed in the xenograft model. The reason for a lack of AKT inhibition *in vivo* is not clear, but could potentially result from compensatory pathway activation by endogenous murine growth factors or cytokines not present in tissue culture media.

### FGFR2 overexpression but not amplification is found in a subset of primary colon cancer and lymph node metastases

**Figure 6:**
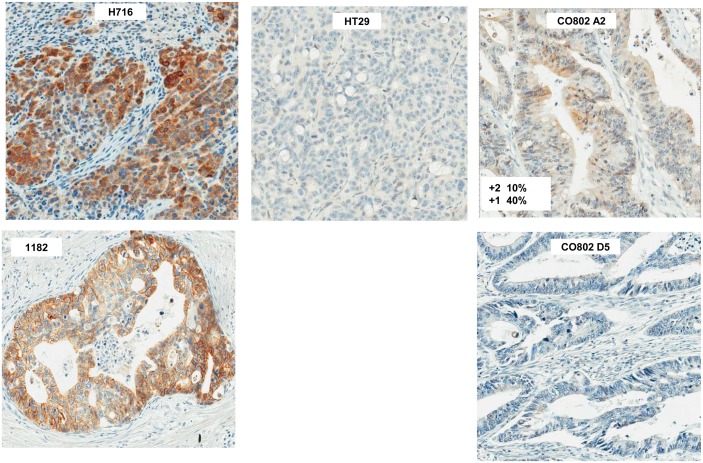
FGFR2 IHC in colorectal cancer arrays. Colon cancer arrays CO702, 802 and CO992 were stained with the H80 FGFR2 antibody (Santa Cruz Biotechnology, Santa Cruz CA) at a 1∶50 dilution on a Ventana Benchmark. Positive controls: NCI-H716, indicates a section from a NCI-H716 xenograft, and 1182 indicates a primary gastric cancer sample harboring *FGFR2* amplification. HT29 is a section from an HT29 xenograft. CO802, A2 indicates a positively staining colorectal cancer section, while CO802 D5 indicates a negatively staining section.

We defined the prevalence of FGFR2 overexpression and amplification in primary colon cancer using immunohistochemistry (IHC) for FGFR2 expression and FISH for *FGFR2* amplification. Because there is precedent for selective *FGFR2* amplification in a metastasis but not in the primary tumor we also analyzed 15 liver and 58 lymph node metastases along with their associated primary tumors. Because previously described C-terminus antibodies do not detect the FGFR2 isoforms in NCI-H716 cells by western blotting or IHC (data not shown), we optimized IHC staining with the N-terminus H80 antibody. We confirmed strong IHC reactivity in the NCI-H716 xenograft and with an *FGFR2* amplified gastric cancer tumor (Figure 6). By contrast HT29 and HCT116 xenograft sections were non-reactive, consistent with their lack of FGFR2 expression (Fig. 1B, western; Figure 6, IHC). We then tested FGFR2 staining in a set of four colorectal cancer tissue microarrays, representing two hundred forty-nine primary samples, fifty eight lymph node metastases, and fifteen liver metastases, and found FGFR2 staining in four primary tumors and one lymph node metastasis (1.5% of all samples). FISH analysis on these arrays revealed no evidence of *FGFR2* amplification (data not shown). We conclude that elevated FGFR2 expression but not amplification can be found in a small subset of colorectal cancers. The lack of widespread staining may in part result from FGFR2 expression heterogeneity, such that a small tumor region with potential positive expression might not be represented by the small array core sample. Examples of this heterogeneity can be seen with Met staining in lung cancer [27] and FGFR2 staining in gastric cancer (B Lutterbach, C. Ware, data not shown). Finally, because the NCI-H716 cell line was derived from an ascites, and has features of endocrine differentiation, it remains possible that FGFR2 overexpression or gene amplification may be present at a higher frequency in colorectal cancers with these histological features.

## Discussion

In this report we reveal that NCI-H716 growth and survival are dependent on the FGFR2 kinase. NCI-H716 is atypical of colon cancer cell lines in several features. First, NCI-H716 cells are wildtype for oncogenes commonly mutated in colon cancer such as *APC, CTNNB1, KRAS, BRAF* or *PIK3CA*, although a *p53* mutation (E224D) is present. The lack of oncogene mutations is consistent with our results that *FGFR2* amplification is the key oncogene driving growth and survival in this cell line. Secondly, this line grows in suspension, which may have been an adaptation for growth as an ascites in the peritoneal cavity. This unattached phenotype suggests a defect in cell adhesion, and in the panel of colon cancer lines only NCI-H716 and RKO cells lacked both EPCAM and CDH1 expression (Figure S8 in File S1). However, retroviral mediated exogenous expression of both CDH1 and EPCAM, individually or in combination, did not enhance cell attachment (data not shown). Finally, NCI-H716 cells have features of endocrine differentiation, ccurring in less than 1% of colon cancer [29], including expression of receptors for serotonin, gastrin and somatostatin, [28] and expression of cytoplasmic core granules [29]. The patient from which the NCI-H716 cell line was derived was also atypical of colon cancer patients. The patient’s young age, 33 years old, is not commonly associated with colon cancer. In fact only 0.1% of all colon cancers occur in patients under 20 years of age, and 1% occur between 20 and 34 years of age [30]. These younger patients often present with advanced-stage disease more commonly than older patients [30] for reasons that are not clear. As described, endocrine cell carcinoma of the colon is rare, and ascites is associated with less than 10% of colon cancer, with both features associated with a poor prognosis [31]. It remains to be determined if *FGFR2* amplification or overexpression are associated with the uncommon histological subsets of ascites or endocrine differentiation.

The mechanism by which *FGFR2* amplification leads to kinase activation could result from dimerization due to high protein expression. As well FGFR2 in NCI-H716 has a loss of C-terminus sequences as seen by lack of reactivity with the C-terminal antibody C20 (data not shown) similar to KATOIII and OCUM2M. In KATOIII and OCUM2M this is due to expression of the c3 splice variant which contributes to transformation [32]. A recent analysis reveals that NCI-H716 cells also harbor the FGFR2 c3 isoform similar to KATOIII, SNU16, and OCUM2M cells [33]. Therefore this splice variant in combination with amplification may be a key factor in kinase activation. We further investigated the potential for autocrine receptor activation in NCI-H716 cells by FGF ligands. Both FGF5 and FGF9 (out of 23 FGFR family ligands) are expressed in NCI-H716 cells (Oncomine, data not shown [25]), but combined treatment with FGF5 and FGF9 blocking antibodies did not block FGFR2 phosphorylation or inhibit NCI-H716 cell growth (data not shown). Therefore autocrine ligand activation does not contribute to activation, and likely the high level of protein overexpression in combination with the splice variant is the key factor for activation.

NCI-H716 cells are similar to other cell lines with growth addiction to receptor tyrosine kinases in that BRAF and RAS are wildtype in these cell lines. It is likely that FGFR2 mediated activation of PI3K and RAS obviates a need to activate these pathways through gene mutation. In NCI-H716 cells the importance of signaling through RAS and PI3K pathways is further supported by the growth inhibition and cell death with a combination of MEK and AKT small molecule inhibitors. By contrast, a MEK inhibitor alone slows growth but does not cause complete stasis, and prominent cell death is not observed. Interestingly neither an AKT inhibitor nor rapamycin inhibited growth alone or in combination. Therefore the AKT survival signaling pathway is critical only when ERK signaling is inhibited. Several targets were linked to ERK signaling, including NFKB and S6RP protein. However, because rapamycin blocks S6RP phosphorylation but did not inhibit growth, it is likely that multiple ERK targets must be inhibited in combination to reproduce the phenotype of MEK inhibition.

We did not observe *FGFR2* amplification in a tissue array of primary colon cancer but did find high expression in a subset of samples. As described above, the patient from which NCI-H716 was derived has features atypical of colon cancer. The available colorectal cancer tissue arrays did not describe the endocrine differentiation status and tumor ascites were not represented. Therefore it remains to be tested whether *FGFR2* amplification may be associated with tumors having endocrine differentiation or with ascites. However, arguing against a high frequency for *FGFR2* amplification in ascites is that other colon cancer cell lines derived from ascites (Colo201, Colo205, and SKCO1) do not harbor *FGFR2* amplification. The oncomine and TCGA colorectal cancer databases did not show *FGFR2* copy gain similar to NCI-H716, but again ascites were not represented (data not shown).

Our results suggest that *FGFR2* amplification is not widespread in common types of colorectal cancer or lymph node and liver metastases. However, it remains possible that defined subsets such as ascites or tumors with endocrine differentiation may have some frequency of amplification. In addition, we observed a small subset of colon tumor harbored FGFR2 overexpression that may result in growth dependency in the tumor cells. The N-terminus antibody that we have identified for IHC is a key reagent to identify FGFR2 overexpression as C-terminus antibodies cannot detect the C-terminal deleted FGFR2 isoforms found in NCI-H716 cells. Finally, our *in vitro* and *in vivo* results suggest that FGFR small molecule inhibitors in clinical development might provide benefit in colorectal tumors harboring FGFR2 overexpression or amplification.

## Supporting Information

File S1 **Combined file of supporting figures. Figure S1. FGFR2 copy gain and overexpression in H716 cells.** A, B. Highly focal copy gain at the *FGFR2* locus at MB 122 in the NCI-H716 cell line and comparison to *FGFR2* amplified gastric cancer cell line KATOIII by SNP aCGH from the Sanger Wellcome Trust Institute. C. Oncomine (Compendia Bioscience, Ann Arbor, MI, USA) database reveals selective *FGFR2* overexpression in NCI-H716 and in *FGFR2* amplified KATOIII and SNU16 cell lines. **Figure S2: FGF2 does not further activate FGFR2 in H716 or colon cancer cell lines.** Lysates were processed as in Figure 1A. “+” indicates addition of 50 ng/ml FGF2 for 15 minutes. **Figure S3: PD173074 is highly selective for FGFR1,2,3.** 100 nM PD173074 was tested for inhibition of the listed kinases on the Ambit kinase binding platform. The platform measures PD173074 binding but not inhibition of kinase activity. The far left column indicates that FGFR1,2,3 bind strongly to PD173074, while DDR1, MKNK1, FLT4, PIK3CB, and CSF1R bind 10–20 fold less tightly. The kinases in the columns on the right bind poorly. Because DDR1 and DDR2 are highly homologous in their kinase domain, and PD173074 did not bind to DDR2, this suggests that binding to DDR1 may be outside the conserved kinase domain. **Figure S4: PD173074 inhibits pFGFR2 and selectively inhibits NCI-H716 growth.** A. FGFR2 phosphorylation in Figure 2 was scanned and quantitated using Image Quant software. IC50 values for inhibition of FGFR2 phosphorylation are indicated. B. Tyrosine kinase inhibitors that lack FGFR2 inhibition do not block growth of NCI-H716 cells. Compounds were used at 1 uM (Gleevec, Tarceva, PD168393), 500 nM (Lapatinib, PHA665752), 100 nM (PD173074) or at 10 ug/ml (anti-IGF1R). NCI-H716 were plated at six thousand cells/well in a 96 well plate, treated with compounds, and processed with Vialight reagent 72 hours later. T = 0 indicates the starting cell number, indicating that PD173074 causes a decrease in starting cell number. **Figure S5: pRSK S359/363 (ERK phosphorylation site) is inhibited by PD173074.** NCI-H716 cells were untreated or treated with 100 nM PD173074 for 2 hours and processed for western blotting as indicated in materials and methods. PhosphoRSK was detected at the ERK phosphorylation site with S359/363 antibody. **Figure S6: L-547 and Rapamycin inhibit Akt and S6RP phosphorylation.** NCI-H716 cells were treated with 1 uM L-547 or 3 nM Rapamycin for 4 hours. Cell lysates were processed for western analysis according to Materials and Methods. **Figure S7 PD173074 causes cell death in NCI-H716 cells.** Cells were treated for 72 hours and photographed. Fragmented cells are consistent with cell death after PD13074 treatment. **Figure S8: E cadherin and EPCAM are not expressed in H716 cells.** Western lysates from Figure 1B were blotted for adhesion molecules E-Cadherin (CDH1) and EPCAM. Arrows indicate NCI-H716 and RKO cells that do not express of CDH1 and EPCAM.
(PPT)
