# Supplementary material for: FGFR2 Is Amplified in the NCI-H716 Colorectal Cancer Cell Line and Is Required for Growth and Survival
Source: PLoS One. 2014 Jun 26;9(6):e98515. doi: 10.1371/journal.pone.0098515 (PMC4072591; doi:10.1371/journal.pone.0098515)
Supplement: File S1 — Combined file of supporting figures. Figure S1. FGFR2 copy gain and overexpression in H716 cells. A, B. Highly focal copy gain at the FGFR2 locus at MB 122 in the NCI-H716 cell line and comparison to FGFR2 amplified gastric cancer cell line KATOIII by SNP aCGH from the Sanger Wellcome Trust Institute. C. Oncomine (Compendia Bioscience, Ann Arbor, MI, USA) database reveals selective FGFR2 overexpression in NCI-H716 and in FGFR2 amplified KATOIII and SNU16 cell lines. Figure S2: FGF2 does not further activate FGFR2 in H716 or colon cancer cell lines. Lysates were processed as in Figure 1A. “+” indicates addition of 50 ng/ml FGF2 for 15 minutes. Figure S3: PD173074 is highly selective for FGFR1,2,3. 100 nM PD173074 was tested for inhibition of the listed kinases on the Ambit kinase binding platform. The platform measures PD173074 binding but not inhibition of kinase activity. The far left column indicates that FGFR1,2,3 bind strongly to PD173074, while DDR1, MKNK1, FLT4, PIK3CB, and CSF1R bind 10–20 fold less tightly. The kinases in the columns on the right bind poorly. Because DDR1 and DDR2 are highly homologous in their kinase domain, and PD173074 did not bind to DDR2, this suggests that binding to DDR1 may be outside the conserved kinase domain. Figure S4: PD173074 inhibits pFGFR2 and selectively inhibits NCI-H716 growth. A. FGFR2 phosphorylation in Figure 2 was scanned and quantitated using Image Quant software. IC50 values for inhibition of FGFR2 phosphorylation are indicated. B. Tyrosine kinase inhibitors that lack FGFR2 inhibition do not block growth of NCI-H716 cells. Compounds were used at 1 uM (Gleevec, Tarceva, PD168393), 500 nM (Lapatinib, PHA665752), 100 nM (PD173074) or at 10 ug/ml (anti-IGF1R). NCI-H716 were plated at six thousand cells/well in a 96 well plate, treated with compounds, and processed with Vialight reagent 72 hours later. T = 0 indicates the starting cell number, indicating that PD173074 causes a decrease in starting cell number. Figure S5: [file pone.0098515.s001.ppt]

## Slide 1
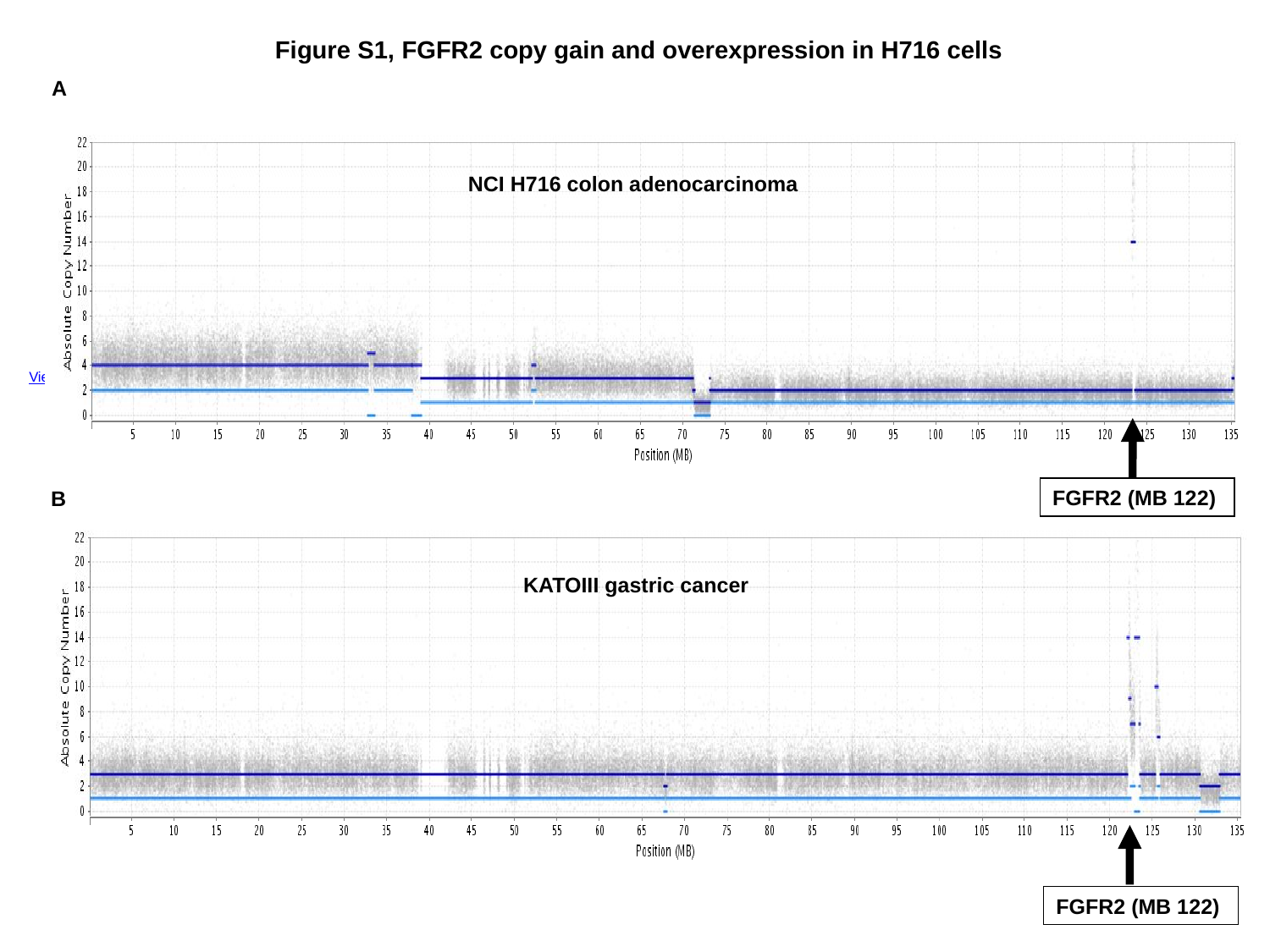

Figure S1, FGFR2 copy gain and overexpression in H716 cells
A
View Key
  NCI H716 colon adenocarcinoma
FGFR2 (MB 122)
B
  KATOIII gastric cancer
FGFR2 (MB 122)

## Slide 2
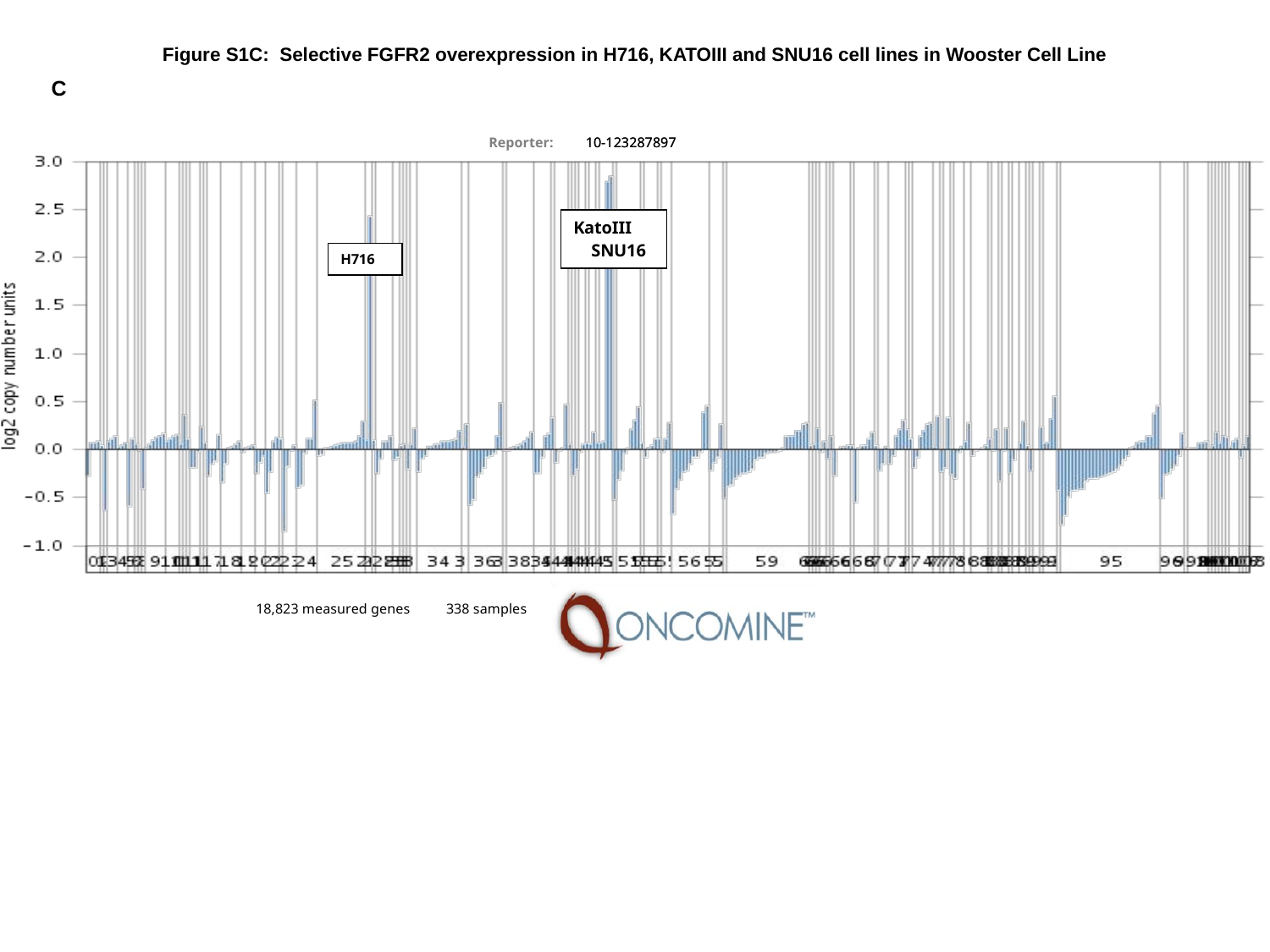

Figure S1C: Selective FGFR2 overexpression in H716, KATOIII and SNU16 cell lines in Wooster Cell Line
C
Reporter:
10-123287897
10-123287897
KatoIII
 SNU16
H716
18,823 measured genes
338 samples

## Slide 3
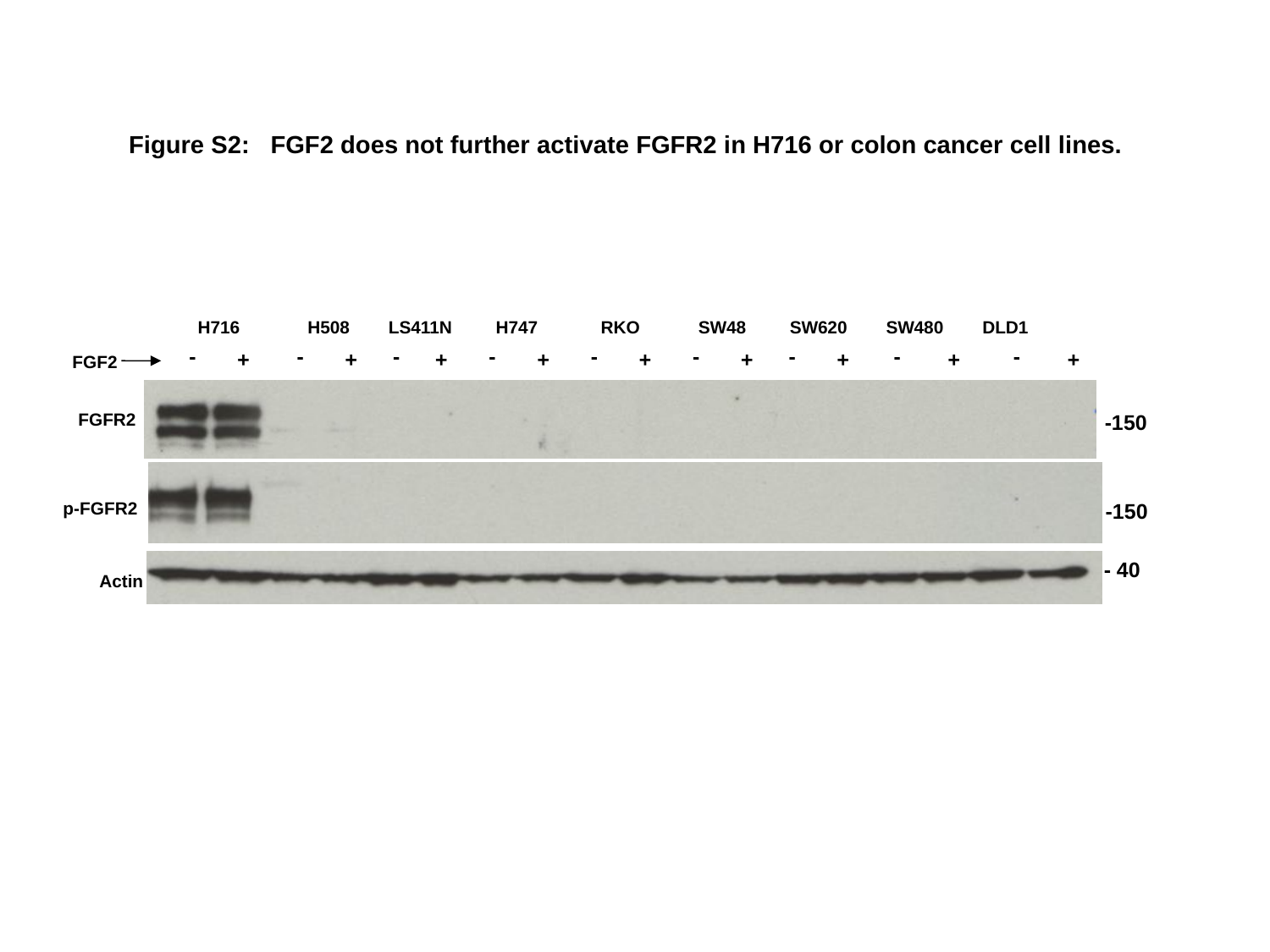

Figure S2: FGF2 does not further activate FGFR2 in H716 or colon cancer cell lines.
 H716 H508 LS411N H747 RKO SW48 SW620 SW480 DLD1
+ - + - + - + - + - + - + - + - + -
FGF2
FGFR2
-150
p-FGFR2
-150
- 40
Actin

## Slide 4
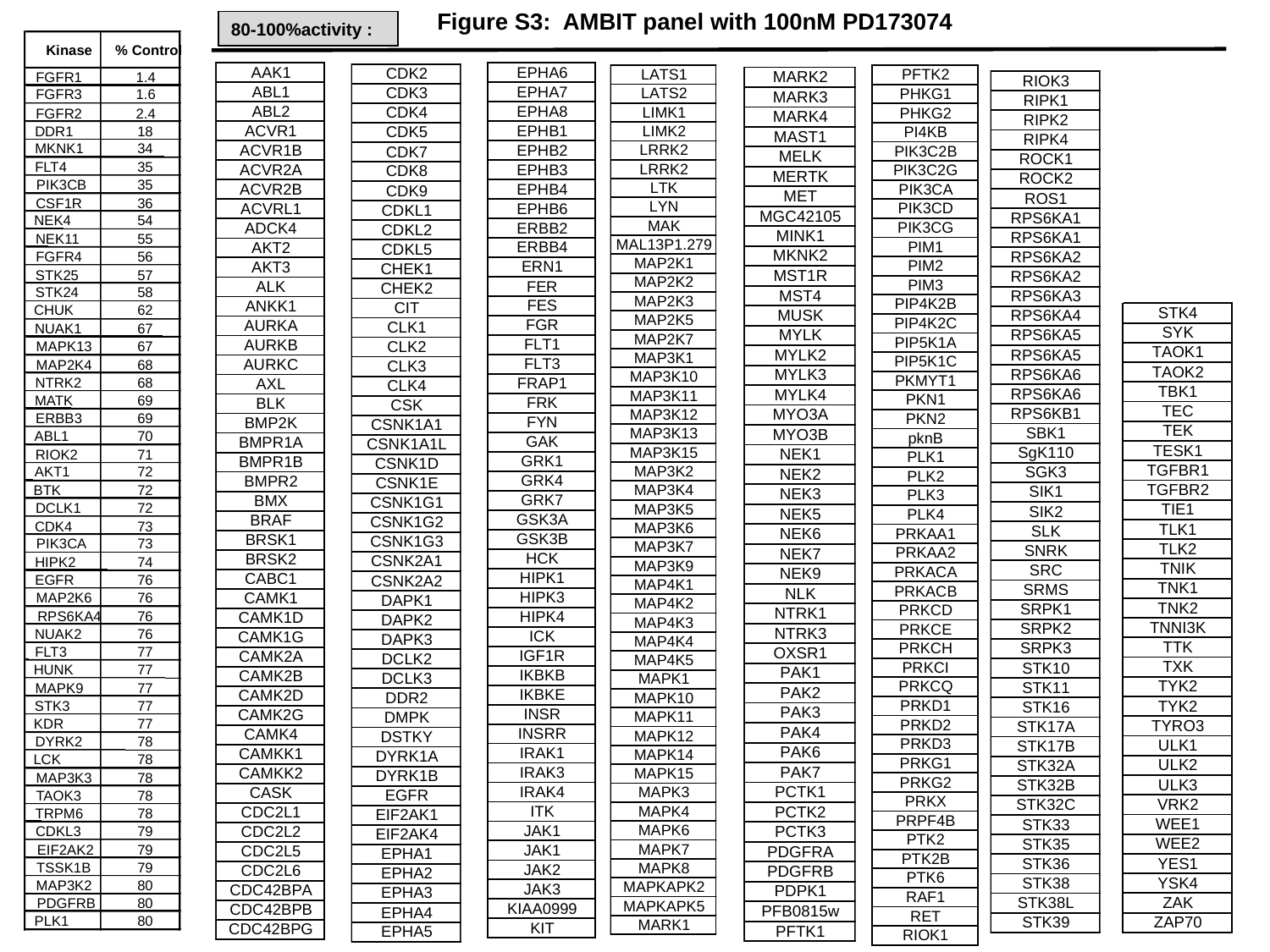

Figure S3: AMBIT panel with 100nM PD173074
80-100%activity :
Kinase
% Control
FGFR1
1.4
FGFR3
1.6
FGFR2
2.4
DDR1
18
MKNK1
34
FLT4
35
PIK3CB
35
CSF1R
36
NEK4
54
NEK11
55
FGFR4
56
STK25
57
STK24
58
CHUK
62
NUAK1
67
MAPK13
67
MAP2K4
68
NTRK2
68
MATK
69
ERBB3
69
ABL1
70
RIOK2
71
AKT1
72
BTK
72
DCLK1
72
CDK4
73
PIK3CA
73
HIPK2
74
EGFR
76
MAP2K6
76
RPS6KA4
76
NUAK2
76
FLT3
77
HUNK
77
MAPK9
77
STK3
77
KDR
77
DYRK2
78
LCK
78
MAP3K3
78
TAOK3
78
TRPM6
78
CDKL3
79
EIF2AK2
79
TSSK1B
79
MAP3K2
80
PDGFRB
80
PLK1
80

## Slide 5
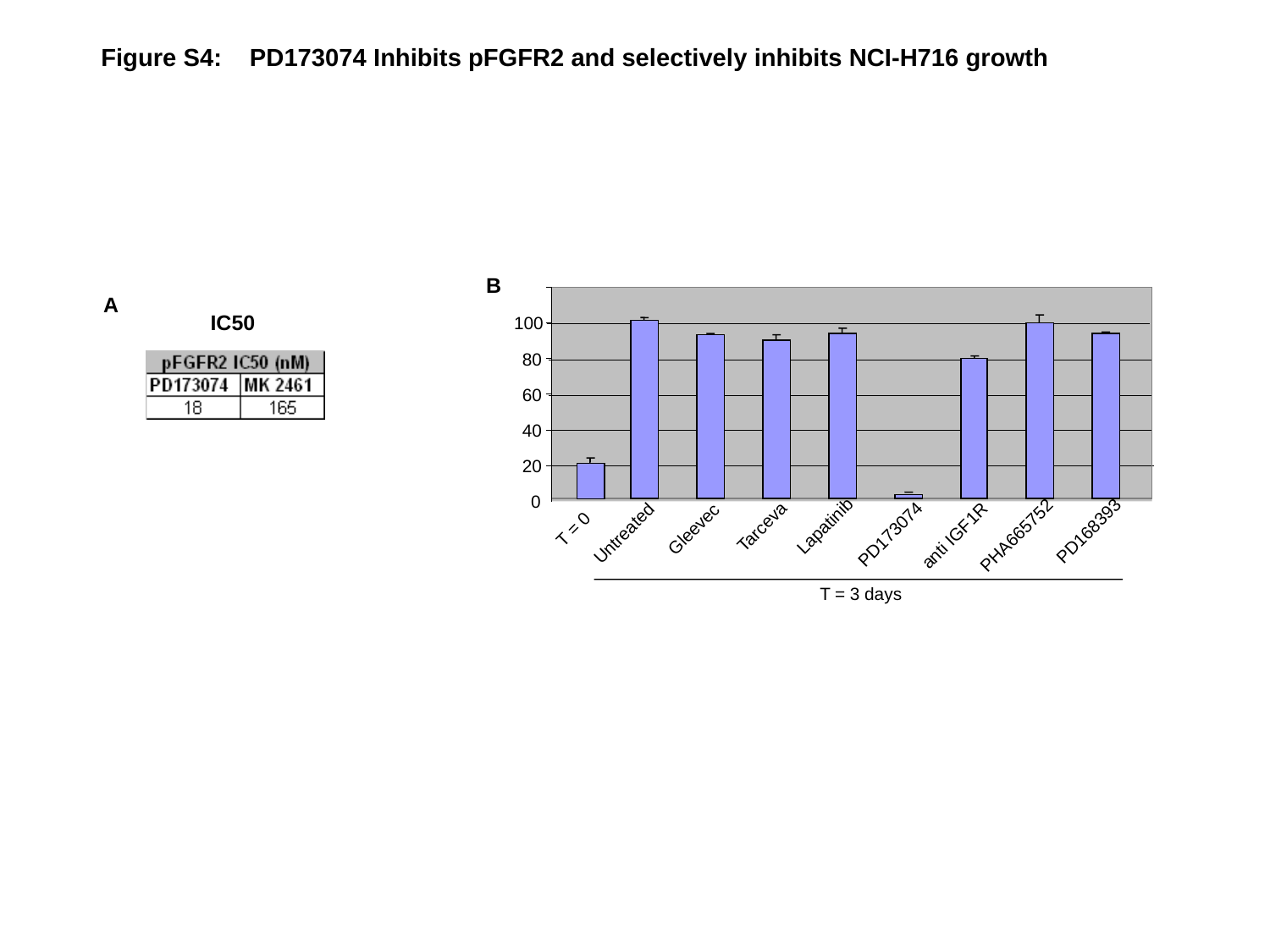

Figure S4: PD173074 Inhibits pFGFR2 and selectively inhibits NCI-H716 growth
B
100
80
60
40
20
0
Lapatinib
Tarceva
Gleevec
T = 0
PD168393
Untreated
PD173074
PHA665752
anti IGF1R
T = 3 days
A
IC50

## Slide 6
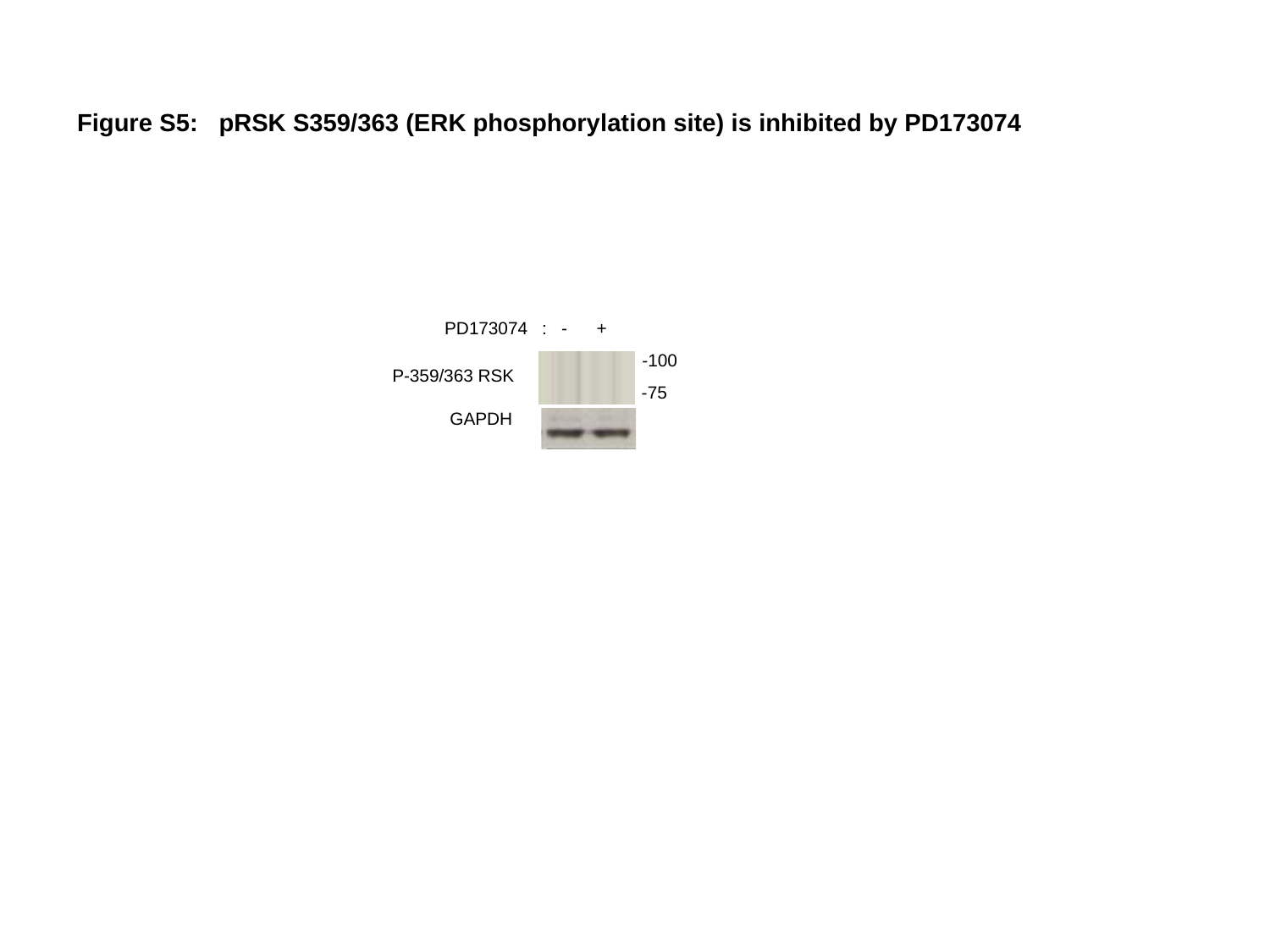

Figure S5: pRSK S359/363 (ERK phosphorylation site) is inhibited by PD173074
PD173074 : - +
-100
P-359/363 RSK
-75
GAPDH

## Slide 7
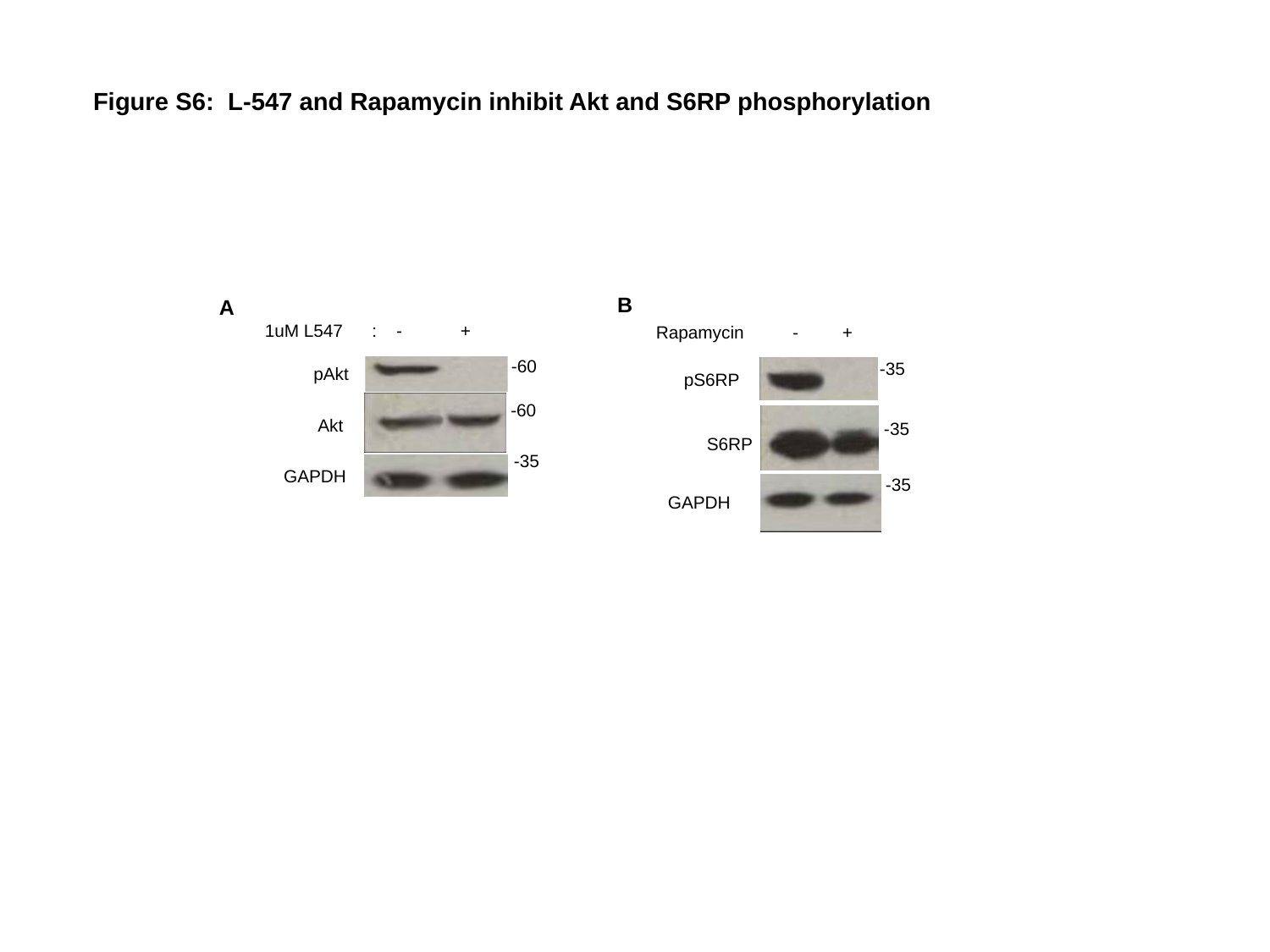

Figure S6: L-547 and Rapamycin inhibit Akt and S6RP phosphorylation
B
A
1uM L547 : - +
Rapamycin - +
-60
-35
pAkt
pS6RP
-60
Akt
-35
S6RP
-35
GAPDH
-35
GAPDH

## Slide 8
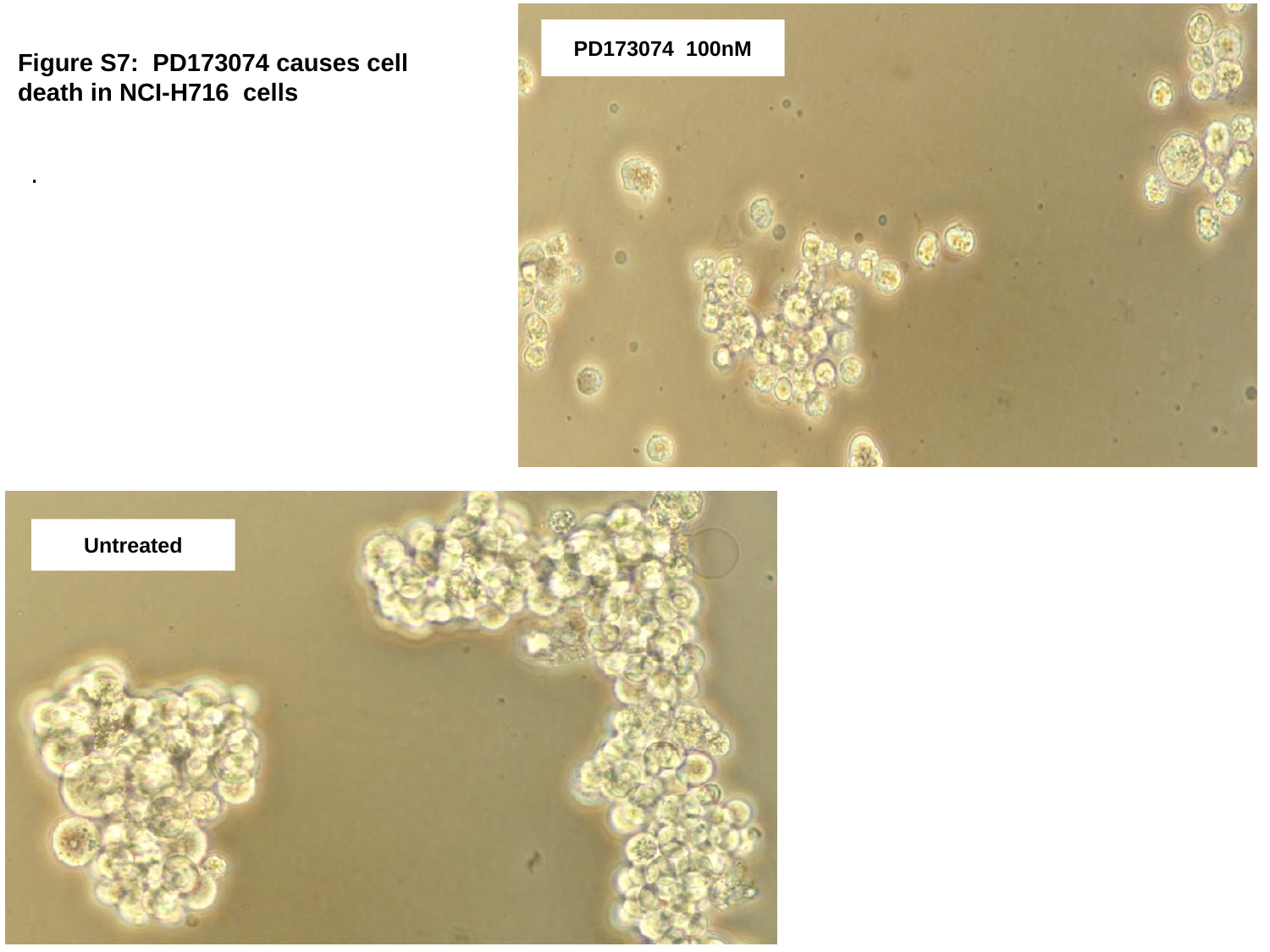

PD173074 100nM
Figure S7: PD173074 causes cell
death in NCI-H716 cells
.
Untreated

## Slide 9
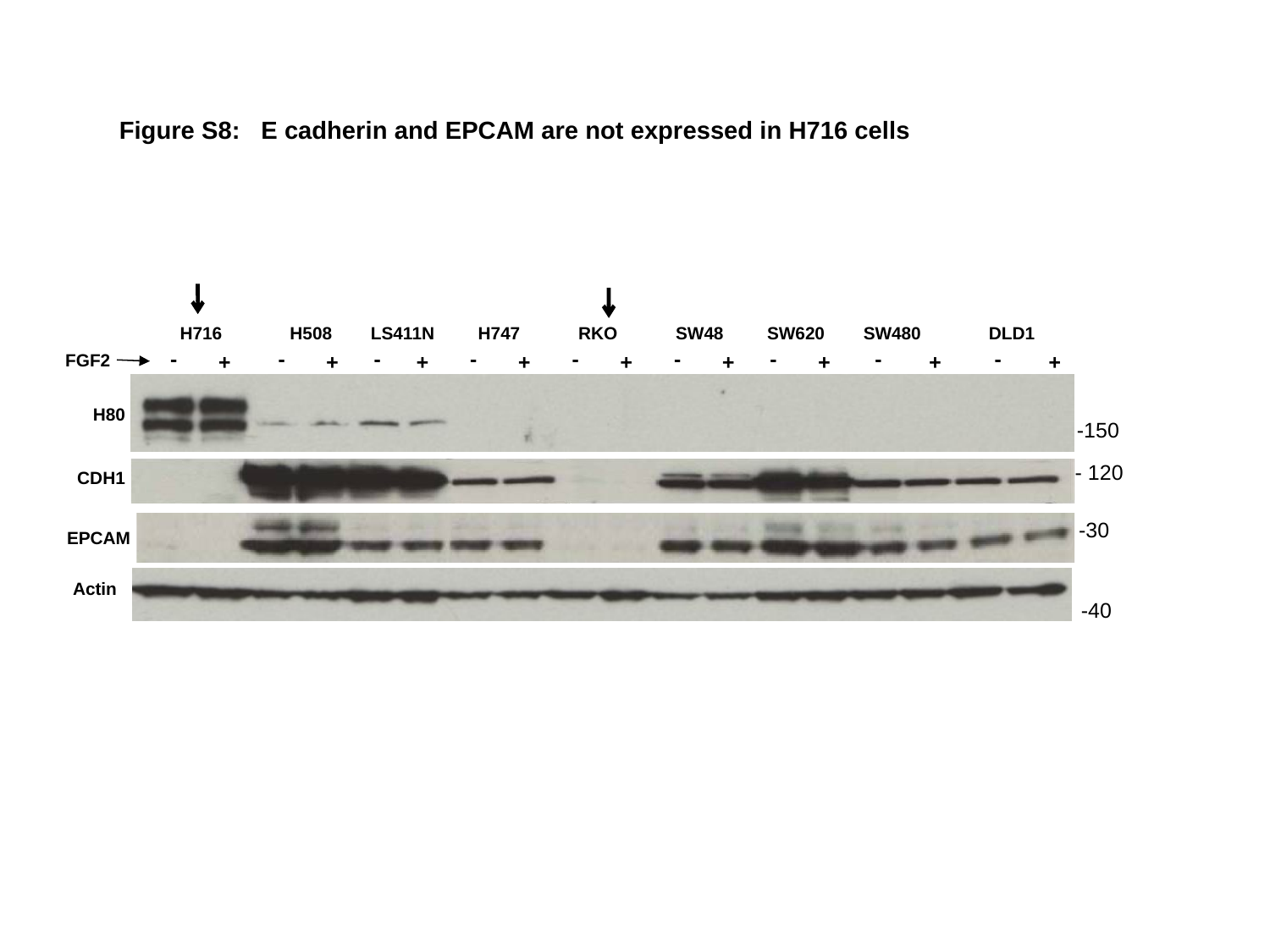

Figure S8: E cadherin and EPCAM are not expressed in H716 cells
 H716 H508 LS411N H747 RKO SW48 SW620 SW480 DLD1
FGF2
+ - + - + - + - + - + - + - + - + -
 H80
-150
-- 120
CDH1
-30
EPCAM
Actin
-40
